# Mechanism of ferroptosis in traditional chinese medicine for clinical treatment: A review

**DOI:** 10.3389/fphar.2022.1108836

**Published:** 2023-01-04

**Authors:** Jiajiao Liu, Guanyin Jiang, Pengfei He, Xing Du, Zhenming Hu, Fuxiang Li

**Affiliations:** ^1^ School of Clinical Medicine, Southwest Medical University, Luzhou, China; ^2^ Orthopedic Laboratory, Chongqing Medical University, Chongqing, China; ^3^ Department of Orthopedics, The First Affiliated Hospital of Chongqing Medical University, Chongqing, China; ^4^ School of Life Science, Beijing University of Chinese Medicine, Beijing, China

**Keywords:** traditional Chinese medicine, ferroptosis, function mechanism, clinical application, clinical disease

## Abstract

Ferroptosis is an iron-dependent regulation of cell death driven by lipid peroxidation, which is intracellularly dependent on iron and independent of other metals, and morphologically, biochemically, and genetically distinct from apoptosis, necrosis, and autophagy. Ferroptosis is closely related to physiological and pathological processes, such as development, aging, and immunity, and it plays an important role in a variety of diseases. In many departments, traditional Chinese medicine plays an increasingly important role in their clinical treatment. In recent years, an increasing number of studies have been conducted on the mechanism of ferroptosis in traditional Chinese medicine. However, the role of ferroptosis in the clinical treatment of traditional Chinese medicine requires further exploration. This article mainly introduces the application of ferroptosis in studies of the mechanism of traditional Chinese medicine to help clinicians understand the current status of traditional Chinese medicine therapy for the treatment of ferroptosis-related diseases.

## Introduction and brief history

Ferroptosis is an iron-dependent and lipid peroxidation-driven process that is significantly different from apoptosis, necrosis, autophagy, and other special forms of programmed cell death at biological and genetic levels (Jiang et al., 2021). Ferroptosis is a completely new form of cell death that was first discovered and reported by Stockwell in 2012 (Dixon et al., 2012). Ferroptosis plays an important role in many physiological and pathological processes, including development, aging, and immunity (Stockwell, 2022). Ferroptosis is inseparable from the occurrence and development of various diseases, including cardiovascular diseases, neurological degenerative diseases, cancers, ischemic organ injuries, endocrine diseases, and respiratory diseases (Hu et al., 2019; Li et al., 2022a; Fang et al., 2022; Lin et al., 2022; Qu et al., 2022; Stancic et al., 2022; Zhang and Li, 2022). Iron accumulation, phospholipid peroxidation, and amino acid, lipid, and sugar metabolism are the most important components of ferroptosis (Jiang et al., 2021). There are two main pathways that induce ferroptosis. First, is the exogenous pathway, which induces ferroptosis by inhibiting cell membrane transporters, such as system Xc- or ferroportin, and activating transferrin and lactoferrin (Li et al., 2020). Second, is the endogenous pathway, which induces ferroptosis by blocking the activation of intracellular antioxidant enzymes, such as glutathione peroxidase GPX4 (Hayashima and Katoh, 2022). The ultrastructural morphological features of ferroptosis include cell membrane tearing and blebbing, mitochondrial atrophy, mitochondrial ridges or even disappearance during ferroptosis, increased membrane density, normal nuclear morphology, and lack of chromatin condensation. Intracellular mitochondria were observed under electron microscopy, and the density of the bilayer film increased. The biological characteristics of ferroptosis include increased reactive oxygen species (ROS), accumulation of iron ions, activation of the mitogen-activated protein kinase system, depletion of glutathione (GSH) by reducing cystine uptake, inhibition of cystine acid/glutamate transporter (system Xc-), and increased reduced acyladenosine dinucleoside phosphate oxidase, thereby releasing mediators, such as arachidonic acid.

Erastin, RSL3, and ferrostatin-1 are key regulators of ferroptosis. Erastin acts on system Xc-, inhibiting the system’s uptake of cystine, resulting in the reduction of endogenous GSH synthesis and the accumulation of intracellular ROS, thereby inducing ferroptosis (Li et al., 2022b). Further, RSL3, which targets GPX4, binds directly to GPX4 and inactivates it, leading to accumulation of lipid peroxides and induction of ferroptosis (Oh et al., 2022). Ferrostatin-1 inhibits erastin-induced accumulation of ROS, thereby inhibiting ferroptosis (Shao et al., 2022). Important markers of ferroptosis are lipid peroxidation markers, including thiobarbituric acid reactive substrates, C11-BODPY fluorescent probe detection, liquid chromatography–mass spectrometry/mass spectrometry lipidomic detection, anti-HNE FerAb Antibody, and anti-malondialdehyde (MDA) addition substrate antibody detection (Yang et al., 2022; Yuan et al., 2022). Intracellular ROS can be detected by fluorescence microscopy, a fluorescence microplate reader, flow detection, and other methods. C11-BODIPY is highly sensitive to free radicals, but does not react with lipid peroxides. Liperfluo is currently the only compound that can specifically detect lipid peroxides. Intracellular ferrous ions can be detected using fluorescence microscopy, confocal microscopy, and a fluorescence microplate reader. Glutathione usually exists in the reduced state GSH, but GSH is converted into the oxidized state, GSSG, under the action of oxidative stress, and the ratio of GSH/GSSG can be considered an important indicator for oxidative stress research (Sun et al., 2022). There are three major inhibitory systems of ferroptosis: the cysteine/GSH/GPX4 axis, NADPH/FSP1/CoQ10 system, and GCH1/BH4/DHFR system (Feng-Jiao et al., 2022; Liu et al., 2022; Wu et al., 2022). The cysteine/GSH/GPX4 axis is an important pathway for ferroptosis, and gene knockout or inhibition of GPX4 activity is an important method to induce ferroptosis. GPX4 is a selenoprotein whose biosynthesis relies on the co-translational integration mechanism of selenocysteine (Feng-Jiao et al., 2022). FSP1 has a potential NADH oxidase function and can inhibit ferroptosis *via* cysteine uptake (Liu et al., 2022). Extramitochondrial ubiquinone can be obtained from the reduction of CoQ10 by FSP1, which can directly capture lipid free radicals or indirectly oxidize α-tocopherol. The guanosine triphosphate cyclohydrolase, GCH1, is a GPX4-independent ferroptosis suppressor gene that cooperates with GPX4 inhibitors to induce ferroptosis (Wu et al., 2022). The purpose of this article is to review the current studies of the ferroptosis in traditional Chinese medicines used in the treatment of various diseases.

## Core mechanisms of ferroptosis in clinical disease

The occurrence and development of various clinical diseases are closely associated with ferroptosis (Table 1). However, many studies on ferroptosis have been conducted at the cellular and animal levels, and there is a lack of clinical validation. Ferroptosis is caused by the accumulation of ROS, which leads to lipid peroxidation, and the accumulation of ROS exceeds the redox content of GSH and GPX4. Ferroptosis is characterized by the accumulation of iron-dependent lipid peroxides at lethal cell levels. The occurrence and execution of ferroptosis depend on the interaction of lipid and iron metabolism, and its sensitivity is regulated by several pathways and processes (Bock and Tait, 2020). Ferroptosis mainly depends on cellular iron ions, and its process includes the accumulation of lipid peroxidation products and ROS, which is an atypical cell death mode. Cell survival is closely related to the relative stability of iron, lipids, and ROS, and when the metabolism is disordered, it causes a fatal blow to the cell (Sun et al., 2021).

**TABLE 1:** Pathways regulating ferroptosis of traditional Chinese medicine in the treatment of various clinical diseases.

Systemic disease	Specific disease	Traditional Chinese medicine	Regulating pathway
Neurological diseases	AD	Ginkgolide B	Nrf2/GPX4 pathway
Cardiovascular diseases	MI/RI	Baicalein	Nrf2/HO-1 pathway
		Shenmai injection	Nrf2/GPX4 pathway
	Ischemic stroke	Naotaifang	System Xc-/Gpx4 pathway
Respiratory diseases	ALL/ARDS	Panaxydol	P62-Keap1-Nrf2 pathway
		Curcumin	Nrf2/HO-1 pathway
Kidney disease	IgA nephropathy	Huaiqihuang granules	ROS-Nrf2/HO-1 pathway
	MsPGN	Huaiqihuang granules	ROS-Nrf2/HO-1 pathway
Endocrine disease	Diabetes	Mulberry leaf extract	System Xc-/GPX4 pathway
Gynecological diseases	EH	Guizhi Fuling capsules	P62-Keap1-Nrf2 pathway
Cancers	TNBC	Shuganning	ROS-Nrf2/HO-1 pathway
	Lung cancer	Tanshinone	P53 pathway
		Artemisinin	System Xc-/Gpx4 pathway
		Puerarin	System Xc-/Gpx4 pathway
	Liver cancer	Artemisinin	System Xc-/Gpx4 pathway
Systemic disease	Specific disease	Traditional Chinese medicine	Regulating pathway

Abbreviations: AD, Alzheimer’s disease; MI/RI, myocardial ischemia-reperfusion injury; ALL, acute lung injury; ARDS, acute respiratory distress syndrome; EH, endometrial hyperplasia; MsPGN, mesangial proliferative glomerulonephritis; TNBC, triple-negative breast cancer.

The main metabolic law of ferroptosis and its classical signaling pathway are mainly caused by the imbalance of three elements, namely, antioxidants, iron, and lipid dynamics. Antioxidants refer to intracellular ROS as an important trigger of ferroptosis, iron is an important factor in ferroptosis, and lipid dynamics refers to lipid metabolism, which is the peroxidation of specific lipid components (Agmon et al., 2018). Iron ions, ROS, and metabolism jointly regulate ferroptosis, and the homeostatic environment of iron ions, metabolic disturbances, and dysregulation of ROS levels are key to ferroptosis. There are three kinds of regulatory mechanisms of ferroptosis. The first kind of pathways regulating iron metabolism including Iron metabolic pathway, ATG5-ATG7-NCOA4 pathway. The second kind of pathways regulated by GSH/GPX4 pathway including P53 pathway, System Xc-/GPX4 pathway and Glutamine metabolic pathway. The third kind of pathways associated with lipid metabolism including Lipid metabolic pathway. However, ferroptosis does not involve the accumulation of ROS, and the accumulation of iron is not exactly similar to ferroptosis. Ferroptosis plays an important role in the occurrence, development, and treatment of several diseases.

## Ferroptosis in neurological degenerative diseases

In animal models, lipid peroxidation and iron accumulation are characteristic of many neurological degenerative diseases, including Alzheimer’s disease (AD), Parkinson’s disease, Huntington’s disease, amyotrophic lateral sclerosis, and multiple sclerosis (Lei et al., 2020). The key role of ferroptosis in neurodegenerative diseases includes the overload of iron ions, lipid peroxidation, and overexpression of ferroptosis-related proteins. The ferroptosis signaling pathway has a bidirectional regulatory effect, which can eliminate pathological cells and maintain a stable state of the body, and it plays an important role in brain and spinal cord injuries (Song and Long, 2020). Ferroptosis inhibitors improve iron homeostasis, lipid metabolism, and redox reactions, resulting in neuronal and functional recovery. Alzheimer’s disease is a devastating neurological degenerative disease, of which there is currently no specific treatment. GPX4 expression is reduced in a mouse model of AD and mice exhibit cognitive impairment (Hambright et al., 2017). Additionally, GSH synthesis is disturbed, and GSH levels are significantly reduced in AD regions of the brain where amyloid plaque density is present (Derry et al., 2020). Ginkgolide B (GB), a terpene lactone derivative from Ginkgo biloba, has neuroprotective functions in various diseases (Derry et al., 2020). Studies have shown that GB can improve cognitive dysfunction in SAMP8 mice by reducing oxidative stress, inflammation, and ferroptosis mediated by Nrf2/GPX4 signaling (Derry et al., 2020). GPX4 inhibitor RSL3 induced ferroptosis and impaired GB-improved cognition in SAMP8 mice. GB alleviates cognitive deficits in AD by reducing oxidative stress, inflammation, and ferroptosis, and exerts beneficial effects on AD by inhibiting ferroptosis (Shao et al., 2021).

## Ferroptosis in cardiovascular diseases

In patients with myocardial ischemia, the heart tissue may be further damaged after recanalization therapy, which is called myocardial ischemia-reperfusion injury. Traditional Chinese medicine (TCM) has the advantages of multiple targets and fewer side effects. Some Chinese medicines have been used clinically for a long time for the treatment of coronary heart disease, angina pectoris, *etc.* Ferroptosis is a novel form of cell death in myocardial reperfusion injury and is characterized by the production of ROS, lipid accumulation, and iron accumulation. Myocardial reperfusion injury is associated with ferroptosis-induced programmed cell death, which activates the PERK-eIF2α-ATF4-CHOP signaling pathway (Peng et al., 2020). Although ferroptosis is related to myocardial ischemia-reperfusion injury, the precise molecular mechanism remains unclear. Myocardial ischemia-reperfusion injury is also associated with myocardial ischemia, hypoxia, and energy metabolism (Lesnefsky et al., 2017). Baicalein was one of the flavonoids with the highest content in *Scutellaria baicalensis*. It has various pharmacological effects, such as anti-inflammatory, bacteriostatic, anti-allergic, anti-viral, cardioprotective, scavenging oxygen free radicals, and low toxicity. Ferroptosis is an important form of cardiomyocyte injury, mainly due to iron-dependent accumulation of lipid peroxidation, and baicalein regulates ferroptosis in cardiomyocytes *via* the Nfr2/HO-1 signaling pathway (Yang et al., 2021a). Ilexsaponin A has many targets against myocardial ischemia-reperfusion injury, including oxidative stress and inflammation. Ilexsaponin A has powerful antioxidant effects, restores mitochondrial function, inhibits myocardial cell apoptosis, and protects cardiac function (Zhang et al., 2017). Shenmai injection alleviates myocardial ischemia-reperfusion through Nrf2/GPX4 signaling pathway-mediated ferroptosis (Mei et al., 2022). Iron accumulation leads to neuronal damage during blood reperfusion, resulting in ischemic stroke in clinical and animal models, and iron chelators reduce ischemic events in animal models of reperfusion injury. Ischemic stroke is a disease caused by ischemic injury, and necrosis is caused by interruption of the blood supply, which in turn leads to impaired neurological function. Stroke is the main cause of disability and death, and it places a huge economic burden on the society. An increasing number of studies have shown that the level of iron ions in the brain tissue of patients with cerebral ischemia is significantly increased, which is closely related to ferroptosis, and that iron-dependent lipid peroxidation can cause cell death (Dixon et al., 2012). Ferroptosis is characterized by intracellular iron overload, GSH deficiency, GPX4 dysfunction, and redox disturbances. In the middle cerebral artery acute brain injury rat model, iron accumulation in rat neurons was observed by Prussian blue staining, and the expression of iron, GSH, ROS, and malondialdehyde (MDA) was detected using kits. Expression of transferrin receptor 1 (TFR1), divalent metal transporter 1 (DMT1), solute carrier family seven member 11 (SLC7A11), and GPX4 were analyzed using immunohistochemistry, quantitative real-time polymerase chain reaction, and western blotting. In rats with acute cerebral ischemia, the expression of TFR1 and DMT1 increased, ROS increased, iron accumulation, lipid peroxidation, MDA, and neurobehavioral equivalence increased, while the expression of SLC7A11 and GPX4 decreased, and the level of GSH and number of Nissl bodies decreased. Additionally, GPX4 expression level was significantly reduced during acute intracerebral hemorrhage, and increasing GPX4 expression can ameliorate secondary neuronal death by ferritin, thereby improving the outcome of intracerebral hemorrhage. Compared with rats in the control group, Naotaifang treatment increased the expression levels of SLC7A11, GPX4, and GSH, as well as the number of Nissl bodies, inhibited ferroptosis through TFR1/DMTI and SCL7A11/GPX4 signaling pathways, thereby improving acute cerebral ischemic injury and neurological function in patients with cerebral ischemia (Lan et al., 2020).

## Ferroptosis in respiratory diseases

Panaxydol is a monomer that has been isolated from ginseng. *In vitro* experiments showed that panaxydol inhibited lipopolysaccharide (LPS)-induced ferroptosis and inflammation in bronchial epithelial cells, BEAS-2B cells. Ferroptosis plays an important role in the progression of acute lung injury (ALI). Lipopolysaccharide induces inflammation in BEAS-2B cells through ferroptosis, and panaxydol ameliorates LPS-induced inflammation by inhibiting ferroptosis (Li et al., 2022a). Panaxydol can upregulate and selectively inhibit the Keap1-Nrf2/HO-1 pathway, and the function of panaxydol in inhibiting ferroptosis disappears (Li et al., 2021). Panaxydol inhibits ferroptosis *via* this pathway and ameliorates LPS-induced ALI. Cigarette smoke extract (CSE) stimulated BEAS-2B cells with intracellular ROS accumulation, lipid peroxidation, and ferroptosis-related proteins, and ROS accumulation and GSH depletion were observed *in vitro*. Cigarette smoke extract stimulates BEAS-2B cell death, higher cytotoxicity, and lower cell viability. The peribronchial inflammatory cell infiltration in cigarette smoke-treated rats was higher than that in the normal group. Cigarette smoke extract increased the levels of interleukin-6 and tumor necrosis factor alpha in rat BEAS-2B cells and bronchoalveolar lavage fluid. In the BEAS-2B cells and rat lung tissue, CSE increased the MDA and iron levels, downregulated GPX4 and ferritin heavy chain levels, and upregulated transferrin receptor levels. Curcumin is the main active component of turmeric, a TCM; it can reduce lipid peroxidation and ferroptosis of airway epithelial cells caused by CSE, reduce lung injury, and improve lung function by activating the Nrf2/HO-1 signaling pathway (Xue and Li, 2018).

## Ferroptosis in kidney disease

Kidney diseases, such as glomerulonephritis, renal tubular injury, and renal vascular disease have a high incidence worldwide, as well as some common complications, such as hypertension and diabetic nephropathy. The pathogenesis and prognosis of kidney diseases are related to oxidative stress, inflammation, mitochondrial damage, ferroptosis, and immune dysfunction. The use of immunosuppressive agents often leads to decreased immunity in patients and damage to the normal structure and physiological function of the kidneys. Huaiqihuang granules, a Chinese medicine, can effectively treat kidney diseases, such as immunoglobulin A nephropathy and mesangial proliferative glomerulonephritis, and Huaiqihuang granules not only enhance patient’s immunity, but also reduce drug toxicity (Liu et al., 2017). Studies have shown that treatment of mouse kidney cells with the chemotherapeutic drug, cisplatin, increased intracellular ROS, decreased GSH, and decreased GSH, which led to decreased GPX4 activity, weakened antioxidant capacity, and induced ferroptosis (Hu et al., 2022). After Huaier polysaccharide intervention, intracellular ROS decreased, GSH activity recovered, the Nrf2/HO-1 signaling pathway was activated, ferroptosis was inhibited, and the antioxidant function of the kidney was improved (Fang et al., 2019). Some Chinese herbal medicines, such as Aristolochiaceae, Hanfangji, and Guanmutong are nephrotoxic. Aristolactam is an active ingredient in some Chinese herbal medicines and is a metabolite of aristolochic acid, which is nephrotoxic (Pu et al., 2016). Studies have shown that aristolochia can inhibit the activity of HK-2 cells in a dose-dependent manner, reduce GSH levels, and increase intracellular iron ions. Aristolactam also increases the level of ferrous ions in the mitochondria and induces mitochondrial damage and mitochondrial membrane density condensation. Ferrostatin-1 can inhibit the accumulation of ROS induced by erastin, thereby inhibiting the occurrence of ferroptosis and reducing the cytotoxicity mediated by aristolactam (Deng et al., 2021). Aristololactam inhibited Nrf2, HO-1, and GPX4 protein expression in a dose-dependent manner.

## Ferroptosis in diabetes

Diabetes is a chronic metabolic disorder that affects several organs. Cell death plays an important role in the development of liver complications in diabetes. Ferroptosis is a form of cell death that is caused by iron-dependent lipid peroxidation. Studies have found that high-glucose feeding and streptozotocin cause β-cell death through the ferroptosis pathway, and the intervention of ferrostatin-1, a ferroptosis inhibitor, can improve β-cell viability, islet morphology, and function. Further research found that in streptozotocin-induced diabetic mice treated with ferrostatin-1, ferrostatin-1 improved serum alanine aminotransferase and triglyceride levels and reduced liver fibrosis (Stancic et al., 2022). Mulberry leaf extract has anti-diabetic effects and can inhibit ferroptosis through the Xc-/GPX4 signaling pathway, thereby affecting blood sugar and islet function and reducing the complications of diabetes (Zhou, 2020).

## Ferroptosis in gynecological diseases

Endometrial hyperplasia is a common gynecological disease, and its main clinical manifestation is abnormal uterine bleeding. Studies have shown that by estradiol-induced mouse endometrial hyperplasia for modeling, the degree of ferroptosis in the endometrial tissue of mice with endometrial hyperplasia is lower than that of normal endometrial tissue, and the ferroptosis inducer, erastin, can improve the mouse endometrial hyperplasia (Fan et al., 2017). Guizhi Fuling capsules inhibited the p62-Keap1-Nrf2 signaling pathway, triggered ferroptosis, and reduced endometrial hyperplasia in a model of endometrial hyperplasia treated with Guizhi Fuling capsules. Estradiol-induced endometrial hyperplasia in mice increases p62 expression, which increases the stability of Nrf2 through the inactivation of Keap1. Guizhi Fuling capsules can reduce p62 expression, promote the accumulation of Keap1, increase the interaction between Keap1 and Nrf2, and increase the degradation of Nrf2. The Nrf2-regulated genes, NQO1, HO1, and FTH1, promote ferroptosis by altering iron metabolism and lipid peroxidation (Zhang et al., 2021a).

## Ferroptosis in cancers

Oxidative stress and chronic iron overload are associated with carcinogenesis. Cancer cells maintain a metabolic balance of ROS within a certain range, and are susceptible to interference with iron metabolism or oxidative stress (Toyokuni et al., 2017). Cancer cells require appropriate catalytic iron for proliferation, thereby utilizing two important antioxidant systems, GSH and Nrf2, to antagonize the persistent oxidative stress generated by the excess iron-mediated Fenton reaction. The Nrf2 is a major transcription factor for antioxidant enzymes, and Nrf2 activation in cancer cells promotes cancer progression and metastasis, and can also develop resistance to chemoradiotherapy (Satoh et al., 2013). Nrf2 is also involved in activating different target genes of iron/GSH metabolism to prevent lipid peroxidation and ferroptosis (Wang et al., 2016). Targeting Nrf2 or inhibiting cysteine/GSH metabolism causes uncontrolled oxidative stress and siderotoxicity, thereby arresting tumor growth and triggering iron-dependent cell death (Wang et al., 2022).

Some small-molecule inhibitors have been shown to induce ferroptosis, thereby inhibiting the growth and metastasis of tumor cells, especially cancers of the liver and pancreas, which is related to their iron richness (Friedmann Angeli et al., 2019). Inhibition of ferroptosis in cancer is not only manifested in the therapeutic effect of cancer but also in reducing the side effects of cancer treatment. Ferroptosis-inducing agents have special effects on a variety of cancers, such as triple-negative breast cancer, diffuse large B-cell lymphoma, renal clear cell carcinoma, hepatocellular carcinoma, *etc.* (Wang et al., 2021a). Ferroptosis inducers can enhance the anti-tumor effect of radiotherapy, and inhibitors of ferroptosis can alleviate the side effects caused by radiotherapy, such as pulmonary fibrosis and myeloid radiation sickness (Ye et al., 2020). Clinical benefits can be achieved for the treatment of cancer by modulating the ferroptosis pathway. The induction of ferroptosis is also resistant to chemoradiotherapy in cancer, and the transcriptional coactivator, Yes-associated protein, is activated in drug-resistant cancer cells, thereby enhancing ferroptosis sensitivity by activating two iron-promoting factors, ACSL4 and TFRC (Yang et al., 2019). Cancer immunotherapy, such as anti-PDL1-mediated tumor immunotherapy, can downregulate SLC7A11 expression to trigger ferritin responses in cancer cells. Combination treatment with an anti-PDL1 antibody and a ferroptosis inducer also showed synergistic anticancer effects in a mouse model (Stockwell and Jiang, 2019; Qin et al., 2022). The activator of ferroptosis strengthens the immune microenvironment link between ferroptosis and cancer (Yang et al., 2021b).

Surgery, radiotherapy, and chemotherapy are currently the main methods for the treatment of gastrointestinal tumors with obvious side effects (Cheng et al., 2022). Betulinic acid has anticancer properties and is a biologically active compound present in the Betula tree. Heme oxygenase-1 (HO-1)-mediated ferroptosis is an anti-tumor therapeutic strategy, and betulinic acid has different effects on HO-1 at different concentrations, thereby decreasing the expression of HO-1 at low concentrations and increasing it at high concentrations of HO-1 expression (Malfa et al., 2019). Numerous studies have shown that HO-1 induces ferroptosis by inducing lipid peroxidation and GSH depletion through an increase in iron-dependent ROS production (Huang et al., 2022). During tumorigenesis and angiogenesis, the expression of HO-1 increases, which leads to cell death through the ferroptosis signaling pathway (Huang et al., 2021). Betulinic acid can significantly reduce the activity of human colon cancer cells, induce an increase in the level of ROS in human colon cancer cells, and exert cytotoxic effects at high concentrations, thereby promoting lipid peroxidation (Chintharlapalli et al., 2011).

The incidence of triple-negative breast cancer without targeted therapy is not low, and the mortality rate is higher than that of other types of breast cancer, accounting for 12–17% of female breast cancer cases, which are prone to ferroptosis (Foulkes et al., 2010). Shuganning, derived from Chinese patent medicine, is widely used as an adjuvant therapy for cancer. Cancer cells have an increased need for iron compared to normal cells, and are more prone to ferroptosis than normal cells. Specific concentrations of Shuganning can induce an increase in ROS levels in triple-negative breast cancer cells, increase the expression of Nrf2 and HO-1, induce ferroptosis, and further inhibit the growth of cancer cells *in vitro* and *in vivo* (Du et al., 2021).

The growth of lung cancer cells is closely associated with ferroptosis. Cisplatin, a commonly used chemotherapeutic drug for lung cancer, can inhibit GPX4, thereby inhibiting ferroptosis (Guo et al., 2018). Tanshinone is an extract obtained from the root of the TCM, Salvia miltiorrhiza. *In vitro*, Tanshinone leads to ferroptosis by inducing p53 upregulation leading to decreased GSH levels and cysteine levels and increased intracellular ROS levels. (Guan et al., 2020). Artemisinin can downregulate the expression of cystine/glutamate transporters and upregulate the mRNA level of the transferrin receptor, thereby promoting ferroptosis in non-small cell lung cancer cells, and its induction of ferroptosis can be inhibited by ferrostatin -1 partial reversal (Zhang et al., 2021b). Puerarin can reduce the damage and inflammatory response in LPS-induced A549 cells and reduce the expression of ROS, MDA, and GSH. At the same time, puerarin reduced the total iron and ferrous ion content in LPS-induced A549 cells and reduced the expression of ferroptosis-related proteins (Zhou et al., 2022).

The incidence of liver cancer is relatively high in Japan, and studies have shown that ferroptosis is related to the treatment and prognosis of liver cancer (Zhang et al., 2022). Artemisinin and its derivatives have various pharmacological effects, such as antimalarial, antiparasitic, antitumor, and autoimmune effects. Studies have shown that artemisinin and its derivatives can lead to the reduction of intracellular GSH, downregulate the protein level of GPX4, further induce ferroptosis, and inhibit the growth of liver cancer cells (Wang et al., 2021b).

Three kinds of pathways can regulate mechanisms of ferroptosis. The first kind of pathways regulating iron metabolism including Iron metabolic pathway, ATG5-ATG7-NCOA4 pathway. The second kind of pathways regulated by GSH/GPX4 pathway including P53 pathway, System Xc-/GPX4 pathway and Glutamine metabolic pathway. The third kind of pathways associated with lipid metabolism including Lipid metabolic pathway.

## Conclusion

**FIGURE 1:**
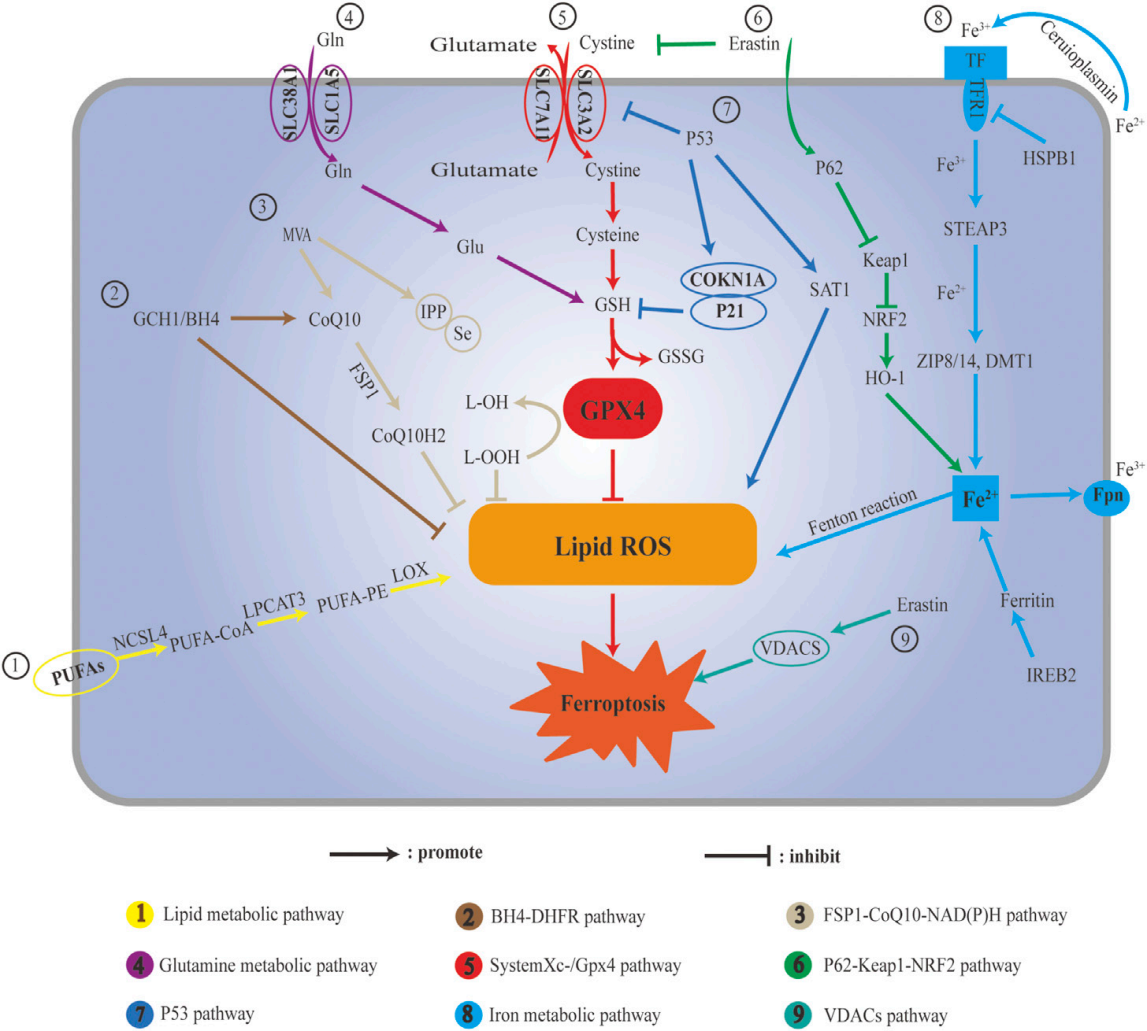
Schematic illustration ferroptosis pathways. **(A)** Lipid metabolic pathway; **(B)** BH4-DHFR pathway; **(C)** FSP1-CoQ10-NAD(P)H pathway; **(D)** Glutamine metabolic: **(E)** c; **(F)** P62-Keap1-Nrf2 pathway; **(G)** P53 pathway; **(H)** Iron metabolic pathway; **(I)** VDACs pathway.

There are an increasing number of studies on ferroptosis, but many mechanisms are not completely clear, and ferroptosis has two sides in the process of disease (Figure 1). Ferroptosis can cause damage to normal cells, leading to disease, and it can eliminate specific pathological states and cells that maintain homeostasis. Stimuli or drugs that induce or inhibit ferroptosis have different effects under different pathological conditions. The regulatory effects of some TCMs and their active components on ferroptosis may be multi-target and multi-pathway. Compared with the induction or inhibition of ferroptosis, TCM has the characteristics of stable structure and safety, and its clinical application requires further research to provide a reference for the development and treatment of TCM for ferroptosis-related diseases.
